# Fear, Anger, and Risk Preference Reversals: An Experimental Study on a Chinese Sample

**DOI:** 10.3389/fpsyg.2017.01371

**Published:** 2017-08-21

**Authors:** Shengxiang She, Iveta Eimontaite, Dangli Zhang, Yan Sun

**Affiliations:** ^1^School of Management, South-Central University for Nationalities Wuhan, China; ^2^Center for Behavior and Decision, Shaanxi University of Technology Hanzhong, China; ^3^Department of Psychology, University of Hull Hull, United Kingdom; ^4^Key Laboratory of Behavioral Science, Institute of Psychology, Chinese Academy of Sciences Beijing, China

**Keywords:** fear, anger, risk preference reversals, framing effect, emotion

## Abstract

Fear and anger are basic emotions of the same valence which differ in terms of their certainty and control dimensions according to the Appraisal Tendency Framework, a theory addressing the relationship between specific emotions, and judgments and choices. Past research based on the Appraisal Theory revealed contradictory results for risky choice decision-making. However, these conclusions were drawn from Western samples (e.g., North American). Considering potential cultural differences, the present study aims to investigate whether the Appraisal Tendency hypothesis yields the same results in a Chinese sample. Our first study explores how dispositional fear and anger influence risk preferences through a classic virtual “Asia Disease Problem” task and the second study investigates how induced fear and anger influence risk preferences through an incentive-compatible task. Consistent with previous research, our results reveal that induced fear and anger have differential effects on risky decisions: angry participants prefer the risk-seeking option, whereas fearful participants prefer a risk-averse option. However, we find no associations between dispositional fear (or anger) and risky decisions.

## Introduction

In recent years, China has seen an increase in the prevalence of cynicism, cyber-bullying, and aggressive behavior in the population. The Annual Report on Social Mentality of China (Wang and Yang, 2011) has suggested that resentment or anger has become the most typical social mentality in current day China. In addition, a public survey of 12 major cities in China (Ouyang, 2010), revealed the severe anxiety that Chinese individuals experience over safety issues such as food, public order, medicine, traffic, and the environment. The emotions of anger and fear easily spread through the present-day internet and social media environment, and may then contribute to or cause real emergencies, intensifying the negative consequences. Hence, fear and anger merit special attention in academic research and policy making.

Since the establishment of the pioneering psychometric model by Fischhoff et al. (1978), researchers have increasingly recognized the important role of emotions in risk perception (Xie et al., 2011). They have found that emotions not only influence people’s cognition, reflected in attention and perception of self and others, but also explain and trigger memories. Researchers in cognitive psychology have considered the effects of affective states in the context of the dual-process theories, which distinguish between a cognitive process that is reliant on analysis versus an emotional one that is reliant on general knowledge structures (for reviews, see Chaiken and Trope, 1999). Although emotions can serve as cues to aid making judgments and decisions, some emotions can interfere with a person’s ability to make rational judgments, and biases their information retrieval and processing, hence, producing a barrier to effective risk communication (Slovic et al., 2004).

Research on judgment and decision making has considered the influence of individual affect. While most of studies in this field have been motivated by a Valence Theory (see Forgas, 1995), relatively few researchers have addressed the influences of specific emotions on judgment and choice. The Valence Theory focuses on the influence of positive emotions compared to negative emotions on decision making (DeSteno et al., 2000). Experimental evidence shows that a negative mood can lead to relatively pessimistic expectations, whereas a positive mood can lead to relatively optimistic expectations (Mayer and Hanson, 1995; Waters, 2008). However, recent studies have provided evidence that contradicts the tempting assumption that all negative emotions fit the valence-congruent patterns; specifically, research into the effects of emotion on attribution, evaluation, and judgments involving risk suggests that anger has distinct effects compared to other negative emotions (Lerner and Tiedens, 2006). An experimental study revealed that fear and anger would result in conflicting effects on a risky choice despite having the same valence (Lerner and Keltner, 2001).

Considering the obvious shortcoming of Valence-based approaches, the more recent Appraisal Theory focuses on specific emotions rather than on global emotion valences (Moors, 2007; Oatley et al., 2011), and therefore accounts for the conflicting effects of different negative emotions like fear and anger on decision making. Fear and anger are the most common negative emotional reactions in daily life and have important implications on decision making under risk (Lerner and Tiedens, 2006). According to Valence Theory, fear and anger should be associated with risk aversion due to their negative valence. However, the Appraisal Tendency Framework (ATF; Han et al., 2007) indicates that fear results in risk aversion while anger leads to risk-seeking choices. The ATF addresses how and why specific emotions carry over from past situations to color future judgments and choices (Han et al., 2007; Lerner et al., 2007). It is assumed that anger and fear are located differently on the dimensions of certainty and control. Past research found that high certainty and individual control are associated with anger, whereas uncertainty and low situational control are associated with fear (Smith and Ellsworth, 1985). Lerner and Keltner (2000, 2001) originally applied the ATF in examining emotion-based differences in judgments and choices involving risk; their results supported the Appraisal-Tendency Hypothesis, that is, fearful people make pessimistic risk assessments, whereas angry people make optimistic risk assessments.

However, will the ATF yield the same results in another cultural context, such as China, a rapidly developing ancient civilization? Although the basic emotions of human beings are universal and people from different cultures have many similar emotional reactions, substantial differences exist among emotional responses, expressions, and concepts in a multi-cultural background (Chen, 2011). In fact, the role of culture on emotional reactivity has been documented in the literature (see Chentsova-Dutton and Tsai, 2010; Chentsova-Dutton et al., 2010). In the United States, expressing anger seems to reflect the degree to which people experience negative events, while in Japan it may reflect the degree to which people feel empowered and entitled. It is believed that anger expression is a complex phenomenon likely motivated by a variety of factors, many of which could be culture-specific (Kitayama et al., 2015). In this case, we must consider the potential cultural differences when using the ATF to explain the judgments and decision making of people in China and to guide policymaking.

Chinese culture has left a deep imprint of “peacefulness” and “reservation” in people’s characters; Chinese people are encouraged to preserve their sanity, by maintaining a peaceful mind, and controlling their emotions in most situations (Wang and Cui, 2008). According to Qiao and Ji (2002), people in Western cultures tend to link the social meanings of behaviors with their emotional experiences, whereas those in Eastern cultures, especially in China, recognize the social meanings of individual behaviors from a social context. Some researchers suggested that members of individualistic cultures are relatively comfortable in expressing their negative emotions, whereas members of collectivistic cultures may feel ashamed to express their negative emotions (Chen, 2011). Take anger as an example; in Western culture people prefer to express their anger, whereas in Eastern culture they prefer to suppress it. Actually, people suppress their anger to avoid the potential effects it might have on their behavior, which complies with the doctrines of Confucius and Mencius in China. The core essence of Confucianism is “benevolence, righteousness, courtesy, wisdom, and trust,” thus Confucianism usually asks people to “deny self and return to propriety” in behaviors. Interestingly, as another important cultural tradition to Chinese people, Buddhism also considers the emotion of anger as one of the Three Poisons that would deeply poison body and mind, so Buddhism asks people to abandon “CHEN” (a religious word similar to anger) and “render good for evil” in their behavior.

Based on these cultural considerations, one might assume Americans would be affected by anger in risky choices, whereas Chinese people might be shielded from the impact of anger on behavior such as making choices under risk. As to the effect of fear on behavior, given that Asians are likely to suppress the expression of their negative emotions, and that expressive suppression reduces risk-taking in Chinese people (Li et al., 2015), the effects of fear on risk choice shown by Lerner and Keltner (2001) might not show up in the present research. However, the merge of Eastern and Western cultures has increased in the last 30 years, and young people in China are deeply influenced by Western culture. Hence, the effects of fear and anger on risky choice preferences are difficult to determine for the current Chinese youth. To our knowledge, the relationship between fear and anger and the risk preferences of Chinese people has not been documented.

The analysis above based on the ATF leads us to hypothesize that angry individuals prefer to make risk-seeking choices while fearful individuals prefer to avoid risk. In the present study, we aim to validate the ATF with Chinese samples by conducting two studies to verify the same hypothesis. First, we examine whether dispositional fear and anger would influence choices under risk by the classic “Asian Disease Problem.” Second, we address whether induced fear and anger would cause the same effect on risk choices by a two-stage choice task with real payoff.

## Study 1: Dispositional Fear, Anger, and Framing Effect

Behavioral economics suggests that the manner in which options are framed alters a decision (Tversky and Kahneman, 1981). In general, individuals tend to be risk-averse in a gain frame – i.e., one in which the task is described in such a way as to emphasize the gains to be made – but tend to be risk-seeking in a loss frame, in which the losses are emphasized. Further, most of respondents become risk seeking when the choices are framed as losses and become risk averse when identical choices are framed as gains. The so-called risk preference reversal is a robust empirical finding, which was explained according to the psychological mechanism of loss aversion defined in the Prospect Theory. However, recent evidence suggests individual differences in emotion have an effect on risky choices and that effect will hold across framing conditions (Lerner and Keltner, 2001). Based on the ATF, the sense of uncertainty and the lack of control associated with fear leads fearful individuals (or those with high dispositional fear) to make risk-averse (certainty enhancing) choices in both gain and loss frames. The sense of certainty and control associated with anger would lead angry individuals (or those with high dispositional anger) to make risk-seeking choices in both gain and loss frames. It is important to note that a Valence Theory would predict that fear and anger should be associated with risk aversion across frames. The present study explored the association between dispositional emotions (anger and fear) and risk choices in order to assess the effect of dispositional anger and fear on risk decision.

### Methods

#### Design

This study used a within-subject design where participants were assigned to both gain and loss frame scenarios in the “Asian Disease Problem” task (as further explained in the Measures and Procedure section). As there were no order effects found in the framing manipulation presentation used by Lerner and Keltner (2001), we did not counterbalance the order of scenarios so that a single online questionnaire could be used in any situation. Furthermore, participants’ dispositional anger and fear (see Measures and Procedure section) were measured after they completed the task.

#### Participants

A total of 372 college students (192 females) participated in this survey. Their age ranged from 18 to 28 years (Mean = 22.45, *SD* = 1.56). To avoid random answers due to incomprehension of the questionnaire, we set up a screening question in the questionnaire after they filled out all the scales: ‘Overall, you have understood the above questions.’ As a result, we eliminated 26 participants who chose the answers ‘A little bit’ or ‘I am not sure’ to filter out rushed answers, resulting in 346 valid samples. This study was carried out in accordance with the recommendations of behavioral experiment guidelines approved by the college Ethics Committee of Guilin University of Technology with written informed consent from all subjects. All subjects gave written informed consent in accordance with the Declaration of Helsinki. The protocol was approved by the college Ethics Committee of Guilin University of Technology.

#### Measures and Procedure

In measuring dispositional fear and anger, the trait scales of Spielberger (1983)’s State-Trait Anxiety Inventory (STAI) and Spielberger (1996)’s State-Trait Anger Expression Inventory (STAXI) are frequently adopted by researchers (e.g., Lerner and Keltner, 2000, 2001). We measured participants’ dispositional anger and fear with the Chinese version of the trait scale of STAI as revised and validated by Tao (2009) and the trait scale of STAXI revised by Li and Qian (1995). The emotion of fear is closely related to anxiety. In psychology, fear is a feeling of doom, unease, or apprehensiveness in response to imminent or immediate danger, while anxiety is a feeling of doom, unease, or apprehensiveness when no danger is present. Therefore, anxiety is the same feeling as fear, but when there is no danger to react to (Öhman, 2010). Anxiety differs from fear in having a fuzzy, potential, or diffuse object which generally emerges from the subject’s own fantasy and imagination. Given that, some studies of fear are actually studies of anxiety, when the object of fear is either fuzzy, potential, or diffuse. For this reason, the subjective emotion ratings as assessed with the trait scale of the STAI and STAXI on a four-point scale (from 1 “never” to 4 “always”) included 20 items about dispositional fear and 10 items regarding dispositional anger.

To measure the risk preference and the framing effect we used the “Asian Disease Problem” task designed by Tversky and Kahneman (1981). In this task, participants were asked to imagine the occurrence of a disease outbreak in a city and to choose between two alternative programs of how to combat the disease. Under the gain frame, participants read the exact scientific estimates of the program outcomes as follows: ‘If Program A is adopted, then 200 people will be saved. If Program B is adopted, then there is a 1/3 probability that 600 people will be saved and a 2/3 probability that no one will be saved.’ Under the loss frame, participants read, ‘If Program A is adopted, then 400 people will die. If Program B is adopted, then there is a 1/3 probability that nobody will die and a 2/3 probability that 600 people will die.’ Choice A implies risk-aversion while choice B implies risk-seeking. Participants had to make choices under both frames. Questionnaires were designed and conducted via survey website Sojump.

### Results

#### The “Asian Disease Problem”

More than half of the participants chose the riskless program A in the gain frame, whereas most of the participants chose the risky program B in the loss frame (**Table 1**). Wilcoxon Sign-rank test suggested significant differences between the choices under the gain and loss frames (*z* = 8.140, *p* ≤ 0.001), thereby confirming the substantial impact of framing on risky choices. Specifically, the difference between choice A and B was not significant within the gain frame (χ^2^ = 3.746, *p* > 0.05), whereas the opposite is the case within the loss frame (χ^2^ = 73.988, *p* < 0.001), thus partly validating the typical risk preference change predicted by prospect theory.

**Table 1:** Choices under different frames.

		Gain	Loss	
A	191	55%	93	27%
B	155	45%	253	73%
Total	346	100%	346	100%

*A represents riskless option and *B* represents risk option.*

#### Dispositional Fear and Anger, and Their Framing Effects

The trait scale of STAXI and the trait scale of STAI were reliable (dispositional fear Cronbach’s α = 0.856, and dispositional anger Cronbach’s α = 0.829). Following this, we produced the dispositional fear (as fearD in **Table 2**) and anger (as angerD in **Table 2**) indices by summing the total scores of all the items of STAI and STAXI, respectively, for each participant as had been done by Tao (2009). The resulting two indices had a significant correlation (Pearson’s *r* = 0.429, *p* ≤ 0.001), which is in line with the findings of Lerner and Keltner (2000).

**Table 2:** Regression of choices on dispositional emotions.

*R*^2^	Factor	*B*	*SE*	Wals	Significance	Exp(*B*)
**Choice under gain frame**						
0.236	FearD	0.005	0.036	0.022	0.883	1.005
	AngerD	-0.058	0.102	0.322	0.570	0.944
	FearD^∗^angerD	0.003	0.004	0.466	0.495	1.003
**Choice under loss frame**						
0.274	FearD	0.047	0.039	1.452	0.228	1.048
	AngerD	0.186	0.114	2.652	0.103	1.204
	FearD^∗^angerD	-0.005	0.004	1.493	0.222	0.995

*FearD is the dispositional fear and *angerD* is the dispositional anger.*

Logistic regressions were performed to ascertain the effects of dispositional fear and anger on the risky choices (risk-seeking = 1, risk aversion = 0) under different frames (**Table 2** for the results).

The significance of the logistic regression model is tested using the Enter method that predicts choice as a function of fear, anger, and their interaction. However, results showed that the risky choices individuals made in either frame were not related to dispositional fear or anger and no interactions were found (*p*’s ≥ 0.103).

We found no evidence in support of our hypothesis in terms of dispositional fear and anger influencing risk decisions. However, we could not conclude that these two emotions have no effects on risk preferences and the following study looking into the relationship between induced anger, fear and risky choices was conducted.

## Study 2: Induced Anger, Fear and Framing Effect

People experience varying emotions in response to different situations in life, and these emotional responses have ‘carryover’ effects on their attitudes and judgments on subsequent events or problems for a while. Emotion induction is a common method to experimentally explore the effects of emotion on various processes. While some researchers have used imagination or memory recall to induce emotions, film clips that contain sounds, images, and texts are a more effective means of inducing affective states in laboratory experiments (Gross and Levenson, 1995). The present study induced emotions by asking participants to watch emotionally charged film clips in order to assess the effect of induced emotions on risk decision.

### Methods

#### Design

A mixed design experiment was designed with the between-subject measures of emotion inducing video watching, and the within-subject measure of making decisions in the gain and loss frame tasks. The success of emotion induction was measured by either a fear (state scale of STAI-Form Y) or anger (state scale of STAXI) questionnaire.

#### Participants

A total of 160 undergraduates (females = 97) who did not take part in Study 1 participated in this experiment. Their age ranged from 18 to 22 years (Mean = 20.38, *SD* = 1.06); 78 and 82 students were randomly assigned to the ‘fear’ group and the ‘anger’ group, respectively. This study was carried out in accordance with the recommendations of behavioral experiment guidelines approved by the college Ethics Committee of Guilin University of Technology with written informed consent from all subjects. All subjects gave written informed consent in accordance with the Declaration of Helsinki. The protocol was approved by the college Ethics Committee of Guilin University of Technology.

#### Measures and Procedure

Emotion induction was performed by asking participants to view 4-min long emotion inducing video clips. The fear group watched a trailer for the horror film named “Sadako reappearance.” This clip had been validated in a pilot study, tested by a 19-item emotional states scale, including fearful, sadness, disgust, happy, anger, and neutrality (“indifferent,” “neutral,” and “unemotional”) (She et al., 2016). Results showed that fear was the unique negative emotion induced by the clip [pre-test Mean = 7.02, *SD* = 4.65, after-test Mean = 12.67, *SD* = 7.05, *t*(56) = 5.86, *p* < 0.001, while all other *p’*s ≥ 0.597].

The anger group was asked to watch a 4-min film clip about children being abused in a nursery. The anger-inducing video clip was from an online news source from October 2012. We did a pretest based on 30 students from the same University using the same 19-item emotional states scale. Mean comparison results showed that anger was changed significantly by the clip [pre-test Mean = 8.92, *SD* = 4.77; after-test Mean = 12.07, *SD* = 7.05, *t*(29) = 4.66, *p* ≤ 0.001], while all other negative emotions were not affected by the clip (*p’*s ≥ 0.256).

It appeared that the experiences of fear or anger varied greatly between participants as reflected in high standard deviations. This is an expected result as individuals’ reactions to the stressors are heterogeneous. Take, for example, horror films; some people may enjoy watching them, while others refuse to watch because they can easily make them feel uncomfortable.

The fear emotion of the participants was measured by 20 items reported on a four-point scale according to the state scale of STAI-Form Y revised by Li and Qian (1995). The anger of the participants was measured by 15 items reported on a four-point scale according to the state scale of STAXI revised by Tao (2009). There are two ways of emotion measurement, one common way is to measure a variety of emotions (such as the application of 19-item emotional states scale in the pre-test of videos) after video watching, and then check whether the target emotions (such as fear) generated in contrast with another baseline group without emotion inducement. The method used in this study is measuring the fear emotion of the fear group and measuring the anger emotion of the anger group without a baseline group. Considering the fact that different participants would experience different levels of emotion, for example, someone has a strong fear while someone has a weak fear, the variability of fear in this group would serve as an independent variable to forecast risk preference. We expected that participants with different levels of emotional inducement would show different risk preferences and therefore we could look into how variation of induced fear or anger influences people’s risk choices.

Participants had to respond to the fear and anger measures, which all used a four-point rating scale where 0 represented “not at all” and 3 “very strongly.” The fear index was calculated as the sum of 20 items of the state scale of STAI-Form Y while the anger index was the sum of 15 items of STAXI. **Table 3** provides the descriptive information of the induced fear and anger indices.

**Table 3:** Description of induced emotions index.

	*N*	Min	Max	Mean	*SD*
Induced anger	82	8.00	39.00	20.6	8.1
Induced fear	78	4.00	55.00	30.2	10.9

Differently from the optimistic risk perception measure used by Lerner and Keltner (2001), participants in our study had to take part in a risky choice task that would determine their remuneration. Many tasks (e.g., the “Asian Disease Problem”) are hypothetical, in which participants are not held accountable for their decisions; thus, their choices might not reflect their true preferences. Considering this potential drawback, we designed a choice task involving a risky decision with attainable monetary outcomes. This part involved two tasks; a choice under gain frame and a choice under loss frame. Before the experiment, the experimenter told the participants that their choice related to their true reward. However, we did not tell them there were two tasks and only the second task determined their payoff until after they had completed the first task. As a result, the participants were led to believe that their choice in the first task would relate to the actual reward, and the purpose of doing so was to ensure they had an incentive for the first choice. In the first task, participants were asked to “please choose between a guaranteed amount of 10 RMB^1^ (about $1.60) (riskless program A) or drawing a ping pong ball from a box (risky program B) with one red ball that pays 30 RMB (about $4.80) and two white balls that pay nothing.” After the first choice task was done, the participants clicked a button to enter the loss frame choice task page that they could not see previously. Before the second choice task, the experimenter explained that the first task will produce no payoffs and emphasized that the second task is independent of the first task and will determine the actual reward. In the second task, all participants were assigned an initial fund of 30 RMB, but were told that they should make a choice to receive their real payoff. They were asked to “please choose either losing 20 RMB (riskless program A) or drawing a ball from the same box containing one red ball and two white balls (risky program B), in which 30 RMB would be deducted for a white ball and no deduction would be applied for a red ball.”

All questionnaires were administered on computers using the Sojump online platform. After the experiment, participants were asked to claim their payments in the designated office the next day. The risk aversion participants, that is, those who chose a certain loss of 20 RMB, received 10 RMB immediately. The risk-seeking ones had to draw a ball from a box containing one red ball and two white balls and received their payoffs based on the draws. The box with the balls was shown and the rules were explained to the participants before the experiments.

### Results

According to **Table 4**, in the fear groups, most of the participants chose the riskless program A compared to the risky program B in the gain frame (76% vs. 24%, χ^2^ = 20.513, *p* < 0.001) whereas there was no significant difference between two programs in the loss frame (49% vs. 51%, χ^2^ = 0.051, *p* > 0.05). On the other hand, most of the participants in the anger groups chose the risky program B compared to the riskless program A in the loss frame (71% vs. 29%, χ^2^ = 14.098, *p* < 0.001) whereas there was no significant difference between two programs in the gain frame (54% vs. 46%, χ^2^ = 0.439, *p* > 0.05). These results suggested a significant preference change as a function of framing. Wilcoxon sign-rank tests suggested significant differences between the choices under the gain and loss frames for fearful participants (*z* = 3.572, *p* < 0.001) as well as for angry participants (*z* = 3.726, *p* < 0.001).

**Table 4:** Number of A and B choices in different frames.

	Fear group		Anger group	
Choice	Gain	Loss	Gain	Loss
A	76%	49%	54%	29%
B	24%	51%	46%	71%
Total	*N* = 78		*N* = 82	

*A represents riskless option and *B* represents risk option.*

#### Fear/Anger and Framing Effects

Logistic regressions were performed to ascertain the effects of induced emotion strength on the risky choices (risk-seeking = 1, risk averse = 0) under the gain and loss frames (**Table 5** for the results). The results imply significant associations between the induced emotion strength and choices under both frames. That is, induced fear strength significantly predicted subsequent the riskless option in both gain (*B* = -0.062, *p* < 0.05) and loss frame (*B* = -0.054, *p* < 0.05), whereas induced angry strength significantly predicted subsequent the risky option in both gain (*B* = 0.079, *p* < 0.01) and loss frame (*B* = 0.100, *p* < 0.01). Note that the data in **Table 4** showed the risky choice as a function of framing in different emotion conditions (i.e., fear and angry), whereas the data in **Table 5** indicated that the induced emotion strength significantly affected subsequent risky choices regardless of frames.

**Table 5:** Logistic regression of choices on induced emotion strength.

Factor	Risky choice	*B*	*SE*	Wals	Significance	Exp(*B*)	*R*^2^
Induced fear	Gain frame	-0.062	0.027	5.080	0.024	0.940	0.262
	Loss frame	-0.054	0.023	5.555	0.018	0.947	0.239
Induced anger	Gain frame	0.079	0.030	6.899	0.009	1.082	0.254
	Loss frame	0.100	0.036	7.626	0.006	1.105	0.226

The above analysis on the relationship between induced emotions and risky choices are based on the overall data, but given that we employed a within-subjects design; we have the advantage to trace the choices of each participant under different frames and detect within-subject preference reversals. Accordingly, we analyzed four patterns of choices under the two frames: ‘A–S: risk averse (gain) – risk seeking (loss),’ ‘S–S: risk seeking (gain) – risk seeking (loss),’ ‘A–A: risk averse (gain) – risk averse (loss),’ and ‘S–A: risk seeking (gain) – risk averse (loss).’ The numbers of participants for the four-choice patterns are shown in **Table 6**. Forty-six percent of the participants in the fear groups exhibited risk-aversion patterns and 43% of the participants in the anger groups exhibited risk-seeking patterns across frames and accounted for the highest percentages in each group. Only a small proportion (*N* = 23, 29%) of the participants exhibited traditional risk preference reversals while a large proportion (*N* = 53, 68% = 22% + 46%) exhibited stable risk preferences across frames in the fear group. The same phenomenon held true in the anger group. Only 23 (28%) of the participants exhibited traditional risk preference reversals while 56 (69%) of the participants exhibited stable risk preferences across frames. The choice pattern between the fear group and the anger group was significantly different (χ^2^ = 10.285, *p* < 0.05). Specifically, the difference between the S–S and A–A pattern was significant within the fear group (χ^2^ = 6.811, *p* ≤ 0.001) but not significant within the anger group (χ^2^ = 3.500, *p* > 0.05).

**Table 6:** Choice patterns across gain and loss frames.

Choice pattern	Fear group		Anger group	
	Number	Proportion	Number	Proportion
A–S	23	29%	23	28%
S–S	17	22%	35	43%
A–A	36	46%	21	26%
S–A	2	3%	3	3%
Total	78	100%	82	100%

*A–S – risk averse (gain) to risk seeking (loss); *S–S* – risk seeking (gain) to risk seeking (loss); *A–A* – risk averse (gain) to risk averse (loss); *S–A* – risk seeking (gain) to risk averse (loss).*

## Discussion

This study explores whether and how fear and anger would influence risk preference based on a sample of Chinese college students. The “Asian Disease Problem” task was used in Study 1 to investigate the effects of dispositional fear and anger on risk preference and a two-stage choice task with attainable monetary outcomes was used in Study 2 to investigate the effects of induced fear and anger. The hypothesis that angry individuals prefer to make risk-seeking choices while fearful individuals prefer to avoid risk was supported by the second study. However, we find no associations between dispositional fear (or anger) and risk decisions in Study 1.

We did not find a significant association between dispositional emotions and risky choices as Lerner and Keltner (2001). A possible explanation is that Chinese people differ from Western people in dispositional fear and anger expression; they are accustomed to separate their dispositional emotions from behaviors. This means that the effects of dispositional emotions on their behaviors are relatively weak compared to Western people, especially when they make decisions in a virtual setting. Thus dispositional emotions might not be a proper variable to forecast behaviors for Chinese people.

The present data in Study 2 demonstrate that emotional state affects risk choice; angry people tend to take risks while fearful people tend to avoid risks. Logistic regression shows that induced fear and anger have a significant negative or positive effect on risk preferences, respectively, that is, fearful and angry individuals tend to make risk aversion or seeking choices across frames. A consistent measurement analysis that trace the choices of each participant across gain and loss frames detecting within-subject preference reversals further confirms the effect of induced emotions on risky choices, i.e., the fearful participants avoid risks whereas angry participants seek risks across frames. The participants experiencing fear or anger were inclined to show stable risk preferences across different frames.

The participants induced with high levels of fear or anger were less influenced by the framing effect and exhibited consistent risk preferences across gain and loss frames. The preference reversals are presumably derived from the affective responses evoked by the decision frames. Recent theoretical research suggests that framing may be akin to an affective heuristic (Slovic et al., 2002), and this argument is supported by evidence from neuroimaging, which shows that decision frames evoke neural activity in brain areas associated with affective processes (De Martino et al., 2006). As a result, highly induced emotions might suppress the affective responses evoked by decision frames.

This main result in this paper supports our hypothesis and is in line with those of Lerner and Keltner (2000, 2001) in terms of induced emotions, which suggests the ATF can be applied in explaining and guiding the decision making of people in China. The second study revealed that fear and anger induced by certain video clips have evident influence on risk choices. The data from this study confirms the relationship between induced emotions and risk preferences. According to the ATF (Han et al., 2007), fear is related to the sense of uncertainty and lack of control, and a fearful individual is inclined to overestimate risk. For instance, fearful residents may boycott an infrastructure project with long-term benefits sightlessly just because of a small probability of a big disaster. Anger is related to the sense of certainty and control. Therefore, an angry individual might become overly optimistic and is more likely to take risks. This person may tend to take excessive risks in making important decisions and therefore suffer great loss. For example, an angry husband makes a bad investment after an intense argument with his wife or after a bitter argument with his coworkers, or an executive officer impulsively chooses a plan that has the potential to exceed the risk tolerance of the organization.

This research has important implications for risk communication for China, which is in a social transitional period and sees increasingly complex social conflicts. The conflicts – such as rich-poor conflicts, labor conflicts, city demolition, medical disputes, moral anomie, and judicial injustices – continue to emerge and impact social mentality through social emotions. Anger and fear are easy to spread under the present-day internet and social media environment, which may evolve into emergencies or further expand the scope of existing emergencies and intensify their negative consequences. Under this context, our research provides an insight to explain the mechanism of some mass incidents from the viewpoint of how negative emotions affect risk judgment. For example, in 2014, the message of building a garbage incineration power plant in the city of Hangzhou raised great concerns among the residents. The situation resulted in protests and vandalism. In this event, the negative emotions – mainly fear and anger – played a key role throughout. First, people became jittery at the mention of garbage incineration because they believe that it potentially affects their health, the environmental quality, and the value of the assets of the surrounding residents. The appraisal of this uncertainty caused fear and further amplified the risk perception. Whereas an investigation in 2012 showed 90.5% of 123 garbage incineration plants have reached the emission standard (Mass protests in response to Hangzhou plans to build waste incineration power plant, n.d.), what the public sees, however, is the 10% unqualified. Obviously, fearful residents are inclined to overestimate the risk of garbage incineration plants, which is confirmed in our experiment. Second, in this event anger drove people to fabricate rumors and riot, which seriously disrupted social order while it brought legal penalties for the offenders. Based on this paper, we argue that angry people would underestimate the possibility of punishment brought on them by their aggressive behaviors, and also strengthen the optimistic attitude of “no punishment if everybody does it.” It suggests that emergency managers and the government should pay attention to the negative emotions of relevant groups in public events. It is important to understand people’s risk perception and take targeted communication programs to further dispose of the physical harm of risks.

A limitation of the current study design was not counterbalancing the scenarios. In order to detect a significant framing effect, researchers generally tend to use a between-subject design rather that a within-subject design to test the preference change, because participants in a within-subject design could often note the connection between two questions and thus make the consistent preference across framings, which would make the observed framing effect insignificant. Obviously, it is harder to capture a significant framing effect with a within-subject design than with a between-subject design. For our case, we found a significant framing effect even with a within-subject design. That means participants still made the different preference across framings even if they noted our framing manipulation. Compared to previous findings, we actually captured a stronger framing effect in the current study. As for the scenario order, we admit that is a potential drawback. Even if Lerner and Keltner (2001) did not find an order effect, future studies should eliminate the limitation of the current study by counterbalancing the study scenarios. In summary, our experiment confirms the validation of the ATF through a sample of college students in China. Our study enriches the literature in emotional decision-making and provides a basis for future explorations based on Chinese samples.

## Author Contributions

Conceived and designed the experiments: YS and SS. Performed the experiments: SS. Analyzed the data: SS and YS. Contributed materials/analysis tools: YS, SS, DZ, and IE. Wrote the paper: YS, SS, and IE.

## Conflict of Interest Statement

The authors declare that the research was conducted in the absence of any commercial or financial relationships that could be construed as a potential conflict of interest.

## Footnotes

1 A regular breakfast costs 3.5 RMB in that university.
